# Identifying pre-post chemotherapy differences in gene expression in breast tumours: a statistical method appropriate for this aim

**DOI:** 10.1038/sj.bjc.6600216

**Published:** 2002-04-08

**Authors:** E L Korn, L M McShane, J F Troendle, A Rosenwald, R Simon

**Affiliations:** Biometric Research Branch, EPN-8128, National Cancer Institute, Bethesda MD 20892, USA; Biometry and Mathematical Statistics Branch, National Institute of Child Health and Human Development, Bethesda MD 20892, USA; Metabolism Branch, Bldg. 10/4N114, National Cancer Institute, Bethesda MD 20892, USA

**Keywords:** cluster analysis, doxorubicin, gene expression, microarray, multiple comparisons, statistical methods

## Abstract

Although widely used for the analysis of gene expression microarray data, cluster analysis may not be the most appropriate statistical technique for some study aims. We demonstrate this by considering a previous analysis of microarray data obtained on breast tumour specimens, many of which were paired specimens from the same patient before and after chemotherapy. Reanalysing the data using statistical methods that appropriately utilise the paired differences for identification of differentially expressed genes, we find 17 genes that we can confidently identify as more expressed after chemotherapy than before. These findings were not reported by the original investigators who analysed the data using cluster analysis techniques.

*British Journal of Cancer* (2002) **86**, 1093–1096. DOI: 10.1038/sj/bjc/6600216 www.bjcancer.com

© 2002 Cancer Research UK

## 

Gene expression profiles of tumour specimens, such as obtained by cDNA microarray experiments, can be studied to address a variety of different scientific aims. One aim is to classify the specimens into newly-formed groups so that the gene expression profile is similar within groups and different between groups; statistical cluster analysis techniques have been used for this type of aim (Eisen *et al*, 1998; Tamayo *et al*, 1999). When the specimens come from pre-specified groups, then there are other possible aims. The aim on which we are focusing in this brief communication is the identification of genes that are expressed differentially between pre-specified groups. Identifying such genes can lead to an understanding of how the groups are different at the cellular-functional level (when the identified genes have known function) and can also lead to clues about the function of identified genes with unknown function. Other possible aims with pre-specified groups include demonstrating global differences between groups using multivariate analysis (without identifying individual genes that are differentially expressed), and developing predictors of group membership (Golub *et al*, 1999; Hedenfalk *et al*, 2001). As we will demonstrate here, it is important to use the appropriate statistical methods to address the particular aim under consideration.

Perou *et al* (2000) studied gene expression profiles measured by cDNA microarrays using specimens from 65 breast tumours from 42 individuals. Among the profiles, data from 20 individuals with specimens taken before and after a 16-week course of doxorubicin chemotherapy were included. Based on a cluster analysis, Perou *et al* (2000) note ‘Gene expression patterns in two tumour samples from the same individual were almost always more similar to each other than either was to any other sample.’ This similarity does not eliminate the equally interesting possibility of finding large and statistically significant differences in pre *vs* post chemotherapy gene expression in the 20 paired specimens. A cluster analysis is not the appropriate statistical analysis for examining this possibility. To find genes that are differentially expressed, we will perform an analysis that is appropriate for this aim. We end with a discussion of the biologic significance of the identified genes.

## MATERIALS AND METHODS

The primary data were obtained at <http://genome-www. stanford.edu/molecularportraits/>and were pre-processed in a standard manner: Data from spots flagged by the original investigators as not useable or which were labelled ‘EMPTY’ were omitted here. In each channel, signal for a spot was calculated as foreground intensity minus background. Spots for which signal was less than 100 in both channels were not used. If the signal was less than 100 in only one channel, the spot was used with the signal set in that channel to 100. The expression ratio was formed as channel 2 divided by channel 1 signal. Ratios were median normalised within each array by dividing the ratios by the median of the ratios for that array. All analyses were performed on log transformed median-normalised expression ratios. Genes for which data were missing from more than half of the 20 paired tumour specimens were eliminated from consideration. This left 8029 genes for analysis.

One statistical method for identifying genes that are differentially expressed is to perform many univariate analyses, testing genes one at a time for differential expression between groups, and then identify the genes which show the most statistically significant differences. In the present application, since there are two groups with paired specimens, an appropriate univariate analysis is a paired *t*-test. One could naively perform 8029 paired *t*-tests, and then identify the genes whose *P*-values were <0.05. There are two problems with this approach. The first is that one would expect 401=0.05×8029 genes to show statistically significant (*P*<0.05) mean group differences even if the expression data were random numbers. The phenomenon of increasing numbers of ‘false positives’ with increasing numbers of hypothesis tests is known as a ‘multiple comparisons problem.’ The second problem with this approach is that standard parametric *t*-tests assume that data are normally distributed. This is not usually a problem in applications with large numbers of samples, but in the present application with small numbers of specimens and where interest is in very small (unadjusted) *P*-values, the normality assumption can be important (Ringland, 1983). We deal with both problems simultaneously by using a step-down permutation approach (Westfall and Young, 1993), an approach that has been used previously to identify differentially expressed genes (Callow *et al*, 2000). This approach does not require normal distributions and controls for the multiple comparisons. In fact, it is less conservative than the frequently used Bonferroni adjustment (Miller, 1981) for multiple comparisons.

Note that the proposed statistical analysis involving 20 pairs of data points for each gene automatically accounts for any noise in the data (e.g., due to mRNA extraction, labelling, hybridisation, and spot-to-spot variations within a microarray). Therefore, it is not necessary to perform replicate microarrays on specimens, replicate clones on each microarray, or provide data on intra- and inter-assay variability measurements; all the data required are the 20 pairs of data points for each gene available on the website. However, the extent to which the sources of random variation are controlled or minimised will affect the power to detect true differential effects. Thus, the quality of the data on the website will affect our ability to detect interesting findings, but the reported statistical significance of findings are accurate regardless of this quality. (This is in contrast to an experiment involving two cell lines, in which one would need data on replicate assays or inter-assay variability to be able to conduct statistical inference.) However, if there are replicate clones or genes spotted on the microarrays, it is of obvious interest to see if they yield similar results. We address this question by examining the differential expression of any genes which have the same name as genes found to have differential expression that is statistically significant.

## RESULTS

Table 1

**Table 1:**
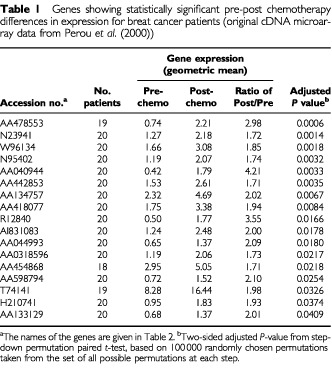
Genes showing statistically significant pre-post chemotherapy differences in expression for breat cancer patients (original cDNA microarray data from Perou *et al* (2000))

and Figure 1

**Figure 1:**
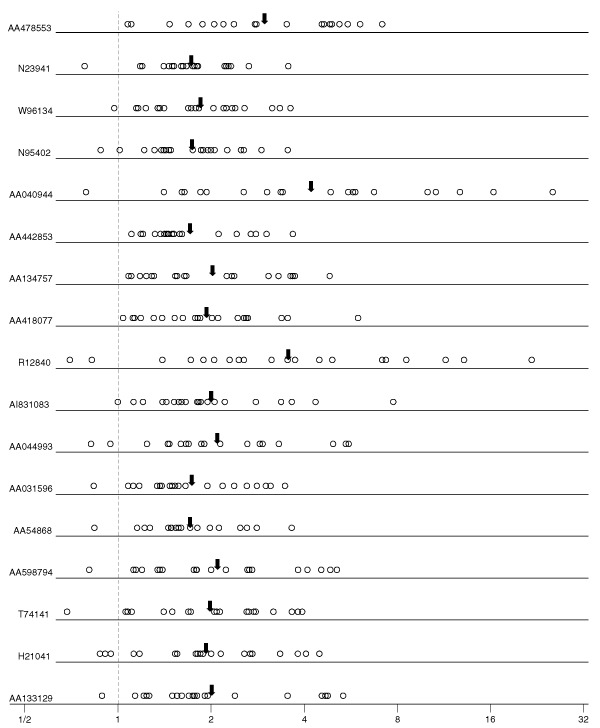
For each gene given in Table 1, plotted points are the ratios of the post-chemotherapy to pre-chemotherapy gene expression ratios for each of 18–20 patients, and arrows are the geometric means of the post/pre ratios.

show the genes identified as being differentially expressed at an adjusted significance level of *P*<0.05 by the step-down permutation paired *t*-test. The *P*-values are adjusted for the multiple comparisons, so that by chance we would expect to identify incorrectly any of the genes as differentially expressed (at adjusted *P*<0.05) less than once in 20. The average expression for each of the 17 identified genes increased after chemotherapy, and all of the specimens for four of the genes showed more expression after the chemotherapy. The names of the genes are given in Table 2

**Table 2:**
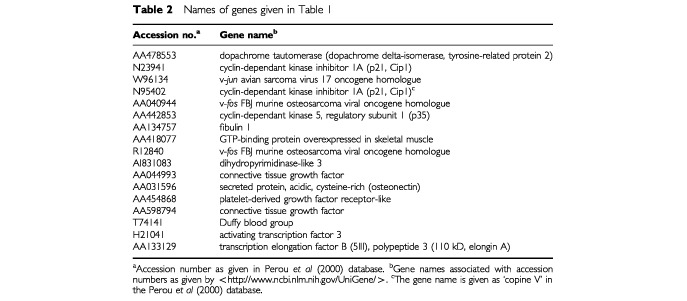
Names of genes given in Table 1

; more information can be found by searching on the accession numbers at <http://www.ncbi.nlm.nih.gov/UniGene/>.

There were no spots in the data base with the same accession number as any of the 17 genes identified in Table 2; we would have expected very close agreement on all gene expression values for such spots. There were nine spots with replicate gene names besides the three pairs of replicate gene names in the identified 17 genes. Gene expressions for all the replicates are displayed in Table 3

**Table 3:**
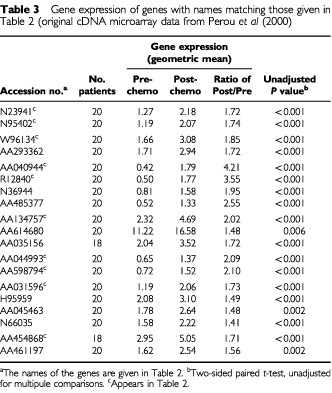
Gene expression of genes with names matching those given in Table 2 (original cDNA microarray data from Perou *et al* (2000)

, along with univariate *P*-values representing the strength of evidence that the clone is differentially expressed (unadjusted for multiple comparisons). The results suggest that the ratio of post/pre gene expression are roughly similar for replicate gene names, and definitely in the same direction (i.e., greater than 1.0). Interestingly, the individual pre and post gene expressions do not always agree well, e.g., AA614680 is expressed at 4–5 times higher levels as compared to the reference sample than the other two clones associated with fibulin 1.

## DISCUSSION

An appropriate statistical analysis has identified genes differentially expressed between pre and post chemotherapy specimens, a task for which cluster analysis is not well suited. The genes identified by this approach reveal important biological insights into the response of breast cancer tumours to doxorubicin treatment. The transcriptional up-regulation of the cyclin-dependent kinase inhibitor p21 reflects the p53-dependent response to doxorubicin induced DNA damage and leads to cell cycle arrest (Vousden, 2000). The up-regulation of c-*fos* and c-*jun* as well as higher expression of genes involved in the stromal reaction and extracellular matrix composition (fibulin 1, connective tissue growth factor, osteonectin) might explain, at least in part, the incomplete response to cytotoxic chemotherapy in some of the tumour cells. In particular, elevated mRNA levels of c-*jun* and c-*fos* have been observed in MCF-7 human breast cancer cells with resistance to doxorubicin as compared to drug-sensitive MCF-7 wild type cells (Daschner *et al*, 1999). Moreover, the adhesion of tumour cells to extracellular matrix proteins may provide a survival signal and confer resistance to chemotherapy-induced apoptosis. In small cell lung cancer, this effect was recently shown to be mediated by the integrin family of receptors (Sethi *et al*, 1999). Therefore, it appears plausible that similar mechanisms might exist in breast cancer.

This communication demonstrates the benefits of providing published microarray data on a website for possible reanalysis by other investigators using different methods or seeking to address different questions.
